# Young Adult Development Indicators for Indigenous and Non-Indigenous People: A Cross-National Longitudinal Study

**DOI:** 10.3390/ijerph192417084

**Published:** 2022-12-19

**Authors:** Elizabeth Doery, Lata Satyen, Yin Paradies, Bosco Rowland, Jennifer A. Bailey, Jessica A. Heerde, Heidi Renner, Rachel Smith, John W. Toumbourou

**Affiliations:** 1 School of Psychology, Burwood Campus, Deakin University, 221 Burwood Hwy, Burwood 3125, Australia; 2 School of Humanities and Social Sciences, Burwood Campus, Deakin University, 221 Burwood Hwy, Burwood 3125, Australia; 3 Monash Addiction Research Centre, Faculty of Medicine Eastern Health Clinical School, Monash University, Clayton 3800, Australia; 4 Social Development Research Group, University of Washington, 9725 3rd Avenue NE, Seattle, WA 98115, USA; 5 Department of Paediatrics, Faculty of Medicine, Dentistry and Health Sciences, The University of Melbourne, Level 2 West, Royal Children’s Hospital, 50 Flemington Road, Parkville 3052, Australia; 6 Murdoch Children’s Research Institute, Royal Children’s Hospital, 50 Flemington Rd, Parkville 3052, Australia

**Keywords:** Indigenous, development, volunteering, community involvement, longitudinal

## Abstract

Worldwide, Indigenous youth face ongoing challenges and inequalities. Increasing our understanding of life course patterns in Indigenous youth will assist the design of strategies and interventions that encourage positive development. This study aimed to increase understanding of resilience and positive development in Indigenous and non-Indigenous youth across Australia and the United States of America. The Australian sample comprised 9680 non-Indigenous and 176 Pacific Islander and Aboriginal and Torres Strait Islander peoples. The USA sample comprised 2258 non-Indigenous and 220 Pacific Islander, Native Hawaiian and Native American/American Indian peoples. Data were used to examine how Indigenous background, volunteering, and community involvement at average age 15 years (Grade 9) predicted five young adult positive development indicators: Year 12 (Grade 12) school completion, tertiary education participation, independent income, paid employment, and intimate relationship formation from age 18 to 28 years. Multilevel regression analyses revealed that while Indigenous youth showed slower increases in positive young adult development over time, when adjusting for socioeconomic disadvantage, there was a reduction in this difference. Moreover, we found that Grade 9 community involvement and volunteering were positively associated with young adult development for Indigenous and non-Indigenous youth. Findings indicate the importance of addressing structural inequalities and increasing adolescent opportunities as feasible strategies to improve positive outcomes for young Indigenous adults.

## 1. Introduction

Historically and contemporarily, Indigenous communities worldwide are disrupted and oppressed by colonial policies and attitudes. Among many destructive consequences, these policies and attitudes perpetuate inequalities between Indigenous and non-Indigenous people, and drive the intergenerational transmission of disadvantage [1,2]. Significant consequences for Indigenous youth include loss of identity and culture, higher rates of suicide, alcohol consumption, and lower school completion rates [3,4].

Adolescence is a fundamental developmental period in life; therefore, further understanding of what may be useful to promote healthy development is crucial [5]. Positive Youth Development (PYD) theory centers on identifying characteristics of optimal young adult development in order to identify strengths, protective factors, and resilience [6]. PYD theories have been articulated into the broader population and recently applied to Indigenous groups [6,7]. The current study explored PYD indicators among Indigenous and non-Indigenous youth in Washington State, USA, and Australia. It also examined whether adolescent community involvement and volunteering influence PYD. Finally, in line with the existing literature [8,9], it probed the role of socioeconomic disadvantage in young peoples’ ability to develop positively.

### 1.1. The Positive Youth Development Theory

PYD theorists argue that given sufficient resources, including high-quality parenting and opportunities to engage with school, family, and communities, young people have the capacity to thrive and develop positively [5,10]. Traditional indicators of positive development include completing education, starting a career, and forming an intimate relationship. More contemporary indicators include emotional, social, and moral competence, self-determination [5,11], and developing an orientation of care for others [12]. However, necessary resources and identified indicators (i.e., what is considered “optimal”) of PYD have been conceptualized and developed largely within European and Western contexts [13]. Most have been developed by societies that have largely colonized traditionally Indigenous-occupied lands. An important, open question is whether these constructs are the most appropriate way to capture optimal PYD indicators, as understood by Indigenous communities. Increasingly, research has begun exploring contributing factors and pathways of PYD across different cultures. Some of this research focuses on protective factors and positive development among Indigenous youth. For instance, Bruner and colleagues [5] suggest that indicators of Aboriginal youth development include empowerment, traditional culture, wellbeing, and resiliency. Other tools to promote development include developing a strong sense of cultural identity and pride [14,15].

A small body of research examines common factors that impact PYD for some Indigenous youth. This literature suggests the importance of strong cultural connectedness in promoting mental health and positive development among Indigenous youth [16,17]. Sanders and colleagues [18] found that receipt of PYD support services (e.g., services which enhance contextual and personal youth resources) were equally beneficial in terms of life satisfaction and education involvement for Māori, Pacific Islander, and Pakeha (non-Indigenous) youth, despite baseline group differences in risk and resilience. These findings suggest that PYD may function similarly for youth across the three cultural backgrounds. Similarly, self-esteem and community engagement [5,19] were identified as important for youth from both Indigenous and non-Indigenous backgrounds. The current study adds to the recent literature by examining whether differences in the development of PYD exist among Indigenous and non-Indigenous youth.

### 1.2. Young Adult Indicators of PYD

Prior research has identified various indicators that promote positive development, including education attainment, strong social relationships, pro-social behavior, pro-social attitudes, and avoidance of violence and alcohol and drug use [19,20,21]. It is also clear that a positive and secure family positively correlates with life satisfaction [22]. Further, Bruner and colleagues [6] identified education involvement as a useful resource to promote positive development among Aboriginal youth in Australia. Other research in Australia has also demonstrated that parents’ education, employment status, and financial circumstances can play a crucial role in the positive development of young Indigenous Peoples [20]. These findings suggest that more generally, education, relationships, employment, and financial circumstances are notable factors in promoting positive development among young people. Based on the existing literature, we examined five indicators of PYD, including Year 12 completion, paid employment, intimate relationship formation, independent income, and tertiary education involvement [18,23,24]. As outlined in the ensuing paragraph, we also examined two protective factors of positive development.

### 1.3. Community Involvement and Volunteering as Protective Factors

Community involvement and volunteering have been highlighted as activities that can promote the positive development of young people [12,25]. The importance of strong community involvement among Indigenous Peoples has been documented in multiple studies, as have the continuing detrimental impacts of colonization on engagement with culture and community among Indigenous youth [26], and resultant costs to the wellbeing of entire communities [27,28]. This literature demonstrates that community involvement can enhance wellbeing, alleviate suffering and grief, and ultimately help to address the impact of socioeconomic disadvantage [20,26]. Strong community involvement can also enhance Indigenous students’ experience and relationship with school [29], and support recovery among Indigenous Peoples experiencing mental illness [30]. Further, it can increase access to wider support and resources, enhancing resiliency, wellbeing, and pro-social outcomes among Indigenous Peoples [30,31]. Crooks et al. [25] also found that engagement in a culturally relevant community program was related to better wellbeing outcomes, increased resiliency, and a more developed cultural identity.

Volunteering has also been established as a predictor of PYD. For example, Hutchinson and colleagues [32] examined the role of care orientation (e.g., volunteering, belief in the moral order, and civic engagement) in predicting psychosocial adjustment in young adulthood. The authors found that involvement in care-oriented activities, including volunteering, may predict positive development (e.g., life satisfaction, trust, social competence) into young adulthood [12,32]. However, there is also research that suggests that there may be some distinctions in protective factors between different cohorts of youth. For example, Musick et al. [33] propose that class, religion, gender, ethnicity, and race may all impact a person’s capacity to volunteer. Consequently, groups unable to volunteer cannot reap the benefits that volunteering provides, which may contribute to disparities. This needs to be considered when comparing levels of volunteer engagement between different cultural groups.

While community involvement has been explored among Indigenous youth and volunteering has been explored among populations more widely, the use of these two constructs to promote PYD have not been examined together across Indigenous and non-Indigenous youth. Therefore, there is a need to better understand the contribution of community and volunteer involvement for Indigenous youth, and to test what dimensions of positive development are enhanced through these factors. To address current gaps in the literature, this study tested the hypotheses that: (1) volunteering and community involvement at age 15 years would predict positive development into young adulthood among participants aged 18 to 28 years living in either Australia or Washington State, USA and, (2) volunteering and community involvement would be similarly predictive of positive development among both Indigenous and non-Indigenous youth.

## 2. Materials and Methods

### 2.1. Design and Participants

This study combined longitudinal data from both the International Youth Development Study (IYDS) and the Young People Our Future (YPOF) study. The combined Australian longitudinal sample from the IYDS and YPOF studies comprised 9680 non-Indigenous and 176 young Pacific Islander and Aboriginal and Torres Strait Islander peoples. The USA sample comprised 2258 non-Indigenous and 220 Pacific Islander, Native Hawaiian and Native American/American Indian youth. These samples are representative of the Indigenous and non-Indigenous populations in Australia and the USA. Specifically, in the USA, the Indigenous populations including the Pacific Islander, Native Hawaiian and Native American/American Indian groups comprise approximately 3% of the overall population and in Australia, the Indigenous populations including the Pacific Islander, and Aboriginal and Torres Strait Islander groups comprise approximately 4% of the overall population. In comparison, in our study, the Pacific Islander, Native Hawaiian and Native American/American Indian comprise approximately 9% of the US sample, and the Pacific Islander and Aboriginal and Torres Strait Islander population comprise approximately 2% of the Australian sample.

The IYDS is a prospective, longitudinal study that aims to understand the etiology of health and social behavior in youth from Victoria, Australia and Washington State, USA. For initial recruitment in 2002, a two-stage clustering approach was used. First, this involved utilizing a probability proportionate to grade-level size sampling procedure to randomly select private and public schools with grades 5, 7 and 9 (ages 11, 13 and 15) [34,35]. Next, one class within each appropriate grade level was randomly selected [35,36]. Full details regarding the IYDS participant and school recruitment processes can be found in McMorris et al. [36]. For the current study, we analyzed data collected when participants were in Grade 9. This was in 2002 for the cohort first recruited in Grade 9, in 2004 for the cohort first recruited in Grade 7, and 2006 for those first recruited in Grade 5. We also analyzed available follow-up data for participants from ages 16 to 29 years, which were drawn from relevant follow-up waves from 2003 to 2018.

The IYDS used a single survey administration protocol in Australia and the USA. The survey was administered by trained survey staff. At the study outset, written parent consent and participant assent were obtained. During adolescence, the survey was administered in school during class time. Following the completion of formal schooling (i.e., in late adolescence and young adulthood), the survey was administered online. Participant consent was obtained for the young adult surveys. To ensure seasonal equivalence in the annual surveys, they were conducted in equivalent seasons (e.g., from May to November in Victoria and from February to June in Washington State).

The YPOF study began in 2017 and recruited Australian adolescents in Grade 8 and Grade 10 (ages 14 and 16), across Government, Catholic and Independent schools, in 28 communities across the states of Victoria, Western Australia, and Queensland [37]. YPOF participants were resurveyed in 2019. A two-stage recruitment process ensured a balanced representation across school types. The YPOF sample was recruited following written parental consent for participation in all waves of the study, and participant consent was again sought at follow up. The survey was primarily delivered online with hardcopy versions available to mitigate technology issues or by school request. For this study, we analyzed Grade 8 (average age 14 years) and Grade 10 (average age 16 years) YPOF data collected in 2017 (wave 1). Combining these cohorts provided a sample of participants who were, on average, in Grade 9 and were, on average, aged 15 years. Follow-up data was taken from YPOF data collected in 2019 (wave 2) when participants were at an average age of 17 years.

### 2.2. Ethics Approval

The Deakin University Human Ethics Committee provided an exemption from ethics approval for this analysis as it draws on de-identified data collected from existing studies that have previously been granted relevant ethics approvals. Original ethics approval was provided by the Deakin University Ethics in Human Research Committee for the collection of YPOF data, and both the Royal Children’s Hospital and The University of Melbourne Human Ethics in Research Committee for the Australian arm of the IYDS. Ethics approval was granted by the University of Washington Human Subjects Institutional Review Board for the USA arm of the IYDS.

### 2.3. Measures

In both studies, longitudinal data were collected using an adapted version of the Communities That Care (CTC) youth survey [35,36]. The CTC survey was reviewed and adjusted to be developmentally appropriate as the samples aged through adolescence and into young adulthood. To achieve data harmonization across studies, relevant YPOF constructs were matched to the closest corresponding IYDS construct. Data from the two corresponding constructs were combined under a newly generated variable. Table A1, Table A2, Table A3 and Table A4 provide details of the variables selected from the different waves of the IYDS and YPOF studies to be combined into the current analysis. Below is a summary of measures and an example question for each construct.

#### 2.3.1. Demographics

Demographic variables included age, sex (male [0] or female [1]), Indigenous status, and state location (Washington, Victoria, Western Australia, or Queensland) of respondents. We recoded state as Australia (1) and Washington (0), and included study YPOF (1) and IYDS (0). To form the Indigenous status variable, the race and ethnicity measure was utilized. Race and ethnicity were measured differently in the USA arm of the IYDS compared to the Australian arm and the YPOF. In the USA, respondents were asked ‘What best describes your racial background?’. Response options included White, Black or African American, American Indian/Native American or Alaskan Native, Asian, Native Hawaiian or other Pacific Islander. In the Australia arm of the IYDS, respondents were asked ‘What do you consider yourself to be?’. Response options included African, Aboriginal or Torres Strait Islander, Spanish, Asian, Pacific Islander, Australian. In the YPOF, participants were asked ‘Are you or your family Aboriginal or Torres Strait Islander?’ (Yes [1] or no [0]). To generate an overall Indigenous variable, participants who identified as American Indian/Native American, Alaskan Native, Native Hawaiian, or other Pacific Islander or Aboriginal and/or Torres Strait Islander were coded as 1; participants who identified as any other ethnicity/race were coded 0 (reference group).

#### 2.3.2. Outcome Variables: PYD Indicators

To evaluate PYD age-trends, indicators were combined by selecting appropriate questions at different longitudinal assessment points after the baseline age of 15. Year 12 completion (coded yes = 1, no = 0) was set to zero for respondents that were still in secondary school or at older ages who did not complete secondary school. An example of how Year 12 completion was measured in the Australian arm of the IYDS was by asking in the post-secondary school years “What is the highest level of secondary school you have completed?” Response options included Year 8 or below, Year 9 or equivalent, Year 10 or equivalent, Year 11 or equivalent, and Year 12 or equivalent. The Australian Year 12 equivalent in the USA is referred to as Grade 12. Students in Year 12 or Grade 12 are generally between the ages of 17 and 18 years. Questions on paid work were included at all age points. An example of how paid work was measured in the YPOF was asking “Are you working (in a paid job)?” and recoded to have two response options (paid work—0 no, 1 yes). Intimate relationships were indicated differently by questions about sexual intercourse (have you ever had sex?) in the secondary school years, and by asking about relationships in the post-secondary years. An example of how intimate relationship was measured in the Australian and USA post-school years in the IYDS was by asking “Are you currently or have you ever been in a romantic or intimate relationship with someone?”. Responses were recoded to indicate current intimate relationship status: no (0, reference group) or yes (1). Income was indicated by earning above $100 per week (recoded yes = 1, no = 0). An example of how income was measured in the Australian and USA arms of the IYDS was by asking “What is your usual take home, weekly income from all sources of support?”, with response options above $100 recoded as income. Tertiary education was assessed as currently studying or previous completion of a certificate (vocational) or degree tertiary course (coded no = 0, yes = 1) and was set to zero while attending secondary school. Across Australia and the USA and different waves of the studies, tertiary education refers to one or more of the following educational qualifications: Technical and Further Education (TAFE), Apprenticeship/Traineeship, University, College, Graduate Diploma/Certificate, Bachelor’s Degree, Postgraduate Degree, Certificate Course or Advanced Diploma/Diploma. An example of how tertiary education was measured in the Australian arm of the IYDS was “What courses or degrees have you studied since secondary school? What is the current status of this course/degree?” Response options included Apprenticeship/Traineeship, Graduate diploma/certificate, Bachelor’s degree, and Postgraduate degree.

#### 2.3.3. Adolescent Predictors

Community involvement was measured using two sub-constructs: opportunity for pro-social involvement, which consisted of five items, and reward for pro-social involvement, which consisted of three items. Responses to all items are measured on a scale from 1 (NO!), 2 (No), 3 (Yes) and 4 (YES!) and averaged to form one measure of community involvement. An example of one item is “My neighbours notice when I am doing a good job and let me know about it.” Volunteering was measured using three items (1) “In the past 12 months have you spent any time doing voluntary work through an organization or group?”, (2) “How often in the past 12 months did you volunteer?”, and (3) “How often have you done volunteer work to help other people, such as helping out as hospital or raising money for charity?”. The three items were merged to form a new variable with participant scores including Never (1), Once (2), Twice (3), 3–4 times (4), and More than 5 times (5).

#### 2.3.4. Control Variables

Parent education was measured as the average of the mother’s and father’s highest level of education (completed—below secondary school (1), secondary school (2), post-secondary education (3)). Government school was measured based on the school sector the respondent attended at baseline: (1) Public or Government school or (0) Independent private, Religious private, Independent, or Catholic. Urbanicity was coded in both Australian datasets using the local government area (LGA) of the respondents’ school at baseline. For the USA arm of the IYDS, urbanicity was based on County. Each County or LGA was then classified as Urban (0), Regional (1), or Rural (2).

### 2.4. Statistical Analysis

All analyses were performed using STATA SE software, Version 15.1 (StataCorp, College Station, TX, USA). Power analyses show the sample is sufficient to identify small effect size differences of 0.35 standard deviation units. For both the IYDS and YPOF data, the relevant variables, including exact age, Indigenous status, community involvement, volunteering, parent education, urbanicity, and school sector, were coded for participants in average Grade 9 (refer to Table 1). Data were organized into a long file format with exact age, follow-up year, and the five adult development indicators coded at each wave that longitudinal data was available starting at age 16 years in the IYDS, and age 17 years in the YPOF. Multilevel mixed-effects models clustered at the individual level were used to analyze age trends in each of the five indicators separately. An additional indicator averaging across the five positive development indicators was calculated at each age to capture overall positive development (refer to Table 2). An interaction term tested whether age trends for the indicators differed for the Indigenous compared to the non-Indigenous sample. Model building progressed in three stages: (1) an unadjusted model including only age and an age x Indigenous interaction term, (2) partially adjusted—model 1 supplemented with demographic and socioeconomic predictors and (3) fully adjusted—model 2 supplemented with adolescent protective factors and their interactions with Indigenous status. Maximum likelihood estimation was used to fit linear models with predictors specified as random effects. The covariance matrix was based on the identity option specifying all variances as equal, and all covariances to be 0. By combining the IYDS and YPOF samples, sample sizes were increased at some ages. However, not all ages were represented in both datasets, creating some data that were missing by design, which can be treated as missing at random (MAR). To handle this, data imputation was used to evaluate bias due to missingness.

## 3. Results

### 3.1. Predictor and Control Variables at Age 15

The demographic information for the Indigenous and non-Indigenous samples in the USA and Australia are summarized in Table 1. Results showed that the average age of all the participants was 14.90 years. In Australia, a higher percentage of Indigenous youth attended a government school (72.27%) compared with non-Indigenous youth (57.87%). There was no observable difference in the USA, with 95.00% of Indigenous youth attending a government school compared to 95.93% of non-Indigenous youth. On average, Indigenous participants in Australia scored higher on the community involvement variable than non-Indigenous participants. In contrast, non-Indigenous participants scored higher on average on community involvement than the Indigenous participants in the USA. Similar findings were identified for the volunteering involvement variable. In Australia, Indigenous participants scored higher, but in the USA, non-Indigenous participants scored higher. In terms of education level, in Australia and the USA, a higher percentage of non-Indigenous participants’ parents had a tertiary education level than the parents of the Indigenous participants. Finally, in Australia, a slightly higher percentage of non-Indigenous participants (58.13%) lived in urban areas compared to Indigenous participants (55.68%). This contrasts with the USA, where a higher percentage of Indigenous participants (65.45%) resided in urban areas compared to the non-Indigenous participants (58.41%).

**Table 1:** Sample description predictor variables at average Grade 9.

	Australia				USA				Total				Total	
Variable	**Indigenous**		**Non-** **Indigenous**		**Indigenous**		**Non-** **Indigenous**		**Indigenous**		**Non-** **Indigenous**		**Whole sample**	
Sample size	176		9680		220		2258		396		11,938		12,334	
Age	**Mean**	**SE**	**Mean**	**SE**	**Mean**	**SE**	**Mean**	**SE**	**Mean**	**SE**	**Mean**	**SE**	**Mean**	**SE**
	14.67	0.08	14.86	0.01	15.12	0.04	15.07	0.01	14.92	0.04	14.90	0.01	14.90	0.01
Community	2.63	0.08	2.59	0.01	2.49	0.06	2.62	0.02	2.55	0.05	2.60	0.01	2.60	0.01
Volunteering	2.29	0.17	2.06	0.02	2.17	0.11	2.63	0.04	2.22	0.10	2.27	0.02	2.17	0.02
	%		%		%		%		%		%		%	
Female	61.36		54.18		51.36		52.83		55.81		53.93		53.99	
Govt school	72.27		57.87		95.00		95.93		87.12		65.07		65.78	
Parent education														
Primary	51.14		40.57		12.27		11.29		29.55		35.03		34.85	
Secondary	30.68		26.22		65.91		50.53		50.25		30.82		31.44	
Tertiary	18.18		33.21		21.82		38.18		20.20		34.15		33.70	
Regionality														
Urban	55.68		58.13		65.45		58.41		61.11		58.18		58.28	
Regional	18.75		19.09		14.55		14.48		16.41		18.22		18.16	
Rural	25.57		22.78		20.00		27.10		22.47		23.60		23.56	

### 3.2. The Age Increase in Positive Adult Development Indicators

The first analysis involved examining each young adult indicator (Year 12, tertiary education, income, paid work, and intimate relationship) separately to determine how indicators change with age when controlling for structural disadvantage, and considering the protectiveness of both community and volunteer involvement. Table 2 (derived from Table 3) shows the change in the positive indicators from age 18 years to age 28 years for the Indigenous sample. Table 2 avoids numerically comparing Indigenous and non-Indigenous youth to highlight Indigenous developmental strengths, in awareness that developmental differences arise from complex causes.

When controlling for structural disadvantage and accounting for the effects of community and volunteering, the growth in tertiary education and intimate relationships were significantly larger among Indigenous youth compared to non-Indigenous youth. Averaged together, the positive indicators among the Indigenous sample increased from 55.54% at age 18 years to 62.32% at age 28 years. In comparison, the positive indicators among the non-Indigenous cohort increased on average from 54.40% at age 18 years to 63.32% at age 28 years. Model fit statistics are presented in Table 3 and reveal the fully adjusted Model 3 was a better fit than the more parsimonious Model 1.

**Table 2:** Positive indicators among Indigenous youth at age 18 and age 28 after controlling for structural disadvantage and other predictors.

Positive Indicator	Age ^1^	Age ^2^
	**18 Years**	**28 Years**
Year 12 completion	22.84% ^	78.83% #
Tertiary education	46.92% ^	60.83% ^
Income	70.03% ^	87.27% #
Paid work	61.62% ^	72.77% #
Intimate relationship	34.36% ^	52.34% ^
Total achieved (of five)	55.54% ^	62.32% #
Standard error	0.02	0.02

^1^ ^ Indicates that Indigenous youth scored higher than the non-Indigenous youth after controlling for structural disadvantage and other predictors (see Table 3). # Indicates that non-Indigenous youth scored higher than the Indigenous youth after controlling for structural disadvantage and other predictors (see Table 3). ^2^ We have included the unadjusted estimates of rates at average age 18 and 28 years for the Year 12 indicator.

Table 2 findings were derived from mixed-effect multilevel regression models performed to estimate the age trend parameter in positive adult development indicators separately, and to predict the average in both Indigenous and non-Indigenous samples across the follow-up years (2003–2019). The results of this analysis are summarized in Table 3. The unadjusted model (Model 1) showed that positive development increased significantly with age, and that this increase was significantly less for Indigenous youth.

**Table 3:** A mixed-effect multilevel regression model predicting average achieved on the five positive indicators.

Predictors	Model 1	Model 2	Model 3
	(Partially Adjusted)		(Fully Adjusted)
	Coefficient	Coefficient	Coefficient
Age	0.047 ***	0.014 ***	0.014 ***
Indigenous	0.098	0.137 **	0.124
Age × Indigenous	−0.009 ***	−0.007 **	−0.007 **
Female	-	0.019 ***	0.018 ***
Follow-up Year	-	−0.030 ***	0.030 ***
YPOF		−0.581 ***	−0.587 ***
Australia		0.206 ***	0.209 ***
School location (comparator-urban)			
Regional	-	0.002	0.001
Rural	-	−0.004	−0.006
Government School	-	−0.039 ***	−0.037 ***
Parent education (comparator-less than secondary)			
Secondary completion	-	0.021 ***	0.020 ***
Post-secondary education	-	0.024 ***	0.020 ***
Community at age 15	-	-	0.012 ***
Indigenous × community at age 15	-	-	0.003
Volunteering at age 15	-	-	0.005 **
Indigenous × volunteering at age 15	-	-	0.002
LL	1440.107	2520.153	2535.828
df	6	15	19
AIC	2868.214	5010.307	−5033.655
BIC	−2823.693	−4899.005	−4892.673

** = *p* < 0.01, *** = *p* < 0.001; LL = Log Likelihood, df = Degrees of Freedom, AIC = Akaike’s Information Criterion, BIC = Bayesian Information Criterion.

Results from Model 2 (see Table 3) revealed that the lower age trend among Indigenous youth remained significantly lower compared to the non-Indigenous youth, but was reduced when accounting for disadvantage and demographic indicators. The variables Female, Parent secondary education, and Parent post-secondary education all significantly increased adult positive development, while Follow-up Year and Government School predicted significant reductions.

#### 3.2.1. Hypothesis Testing

The results from Model 3 revealed that community and volunteering involvement were associated with a significant increase in positive adult development. This model further showed that the interaction between community involvement and positive development, and the interaction between volunteering and positive development, were not significantly different for Indigenous and non-Indigenous youth. 

#### 3.2.2. Predictors 

Finally, model 3 revealed that several predictors remained significant from Models 1 and 2. These included Age, Female, Follow-up Year, YPOF, Australia, Government School, Parent secondary education, and Parent post-secondary education. Therefore, our models demonstrated that while there was a difference in adult positive development between the two groups of youth, it increased for both the Indigenous and non-Indigenous youth and that this growth in development was impacted by several predictors.

## 4. Discussion

This study reports the first longitudinal, cross-national study that examined age trends in young adult development indicators among Indigenous and non-Indigenous youth. Although it was identified that Indigenous youth showed slower increases in positive young adult development over time, when adjusting for socioeconomic disadvantage, there was a reduction in this difference. These findings are consistent with PYD theory [5,23], and with prior studies that found Indigenous developmental disadvantage to be explained by higher socioeconomic disadvantage [8,9] These findings further highlight the continuing adverse impacts of colonialism and social and economic exclusion on the developmental potential of Indigenous individuals.

In line with the hypotheses, we found that community involvement and volunteering were positively associated with young adult development for Indigenous and non-Indigenous youth. Effects were significantly larger in the Australian samples relative to Washington State. As per our second hypothesis, volunteering and community involvement were similarly predictive of positive development among both Indigenous and non-Indigenous youth (Table 3, Model 3). However, we still observed some developmental disadvantage among the Indigenous cohort, despite controlling for structural disadvantage and considering community and volunteer involvement. This may be explained through other experiences including racism, which the current study did not measure. Experiences of racism remain prevalent today, breeding inequalities between Indigenous and non-Indigenous youth in both Australia and the USA [38,39].

The two development markers we identified with a greater increase among the Indigenous sample were intimate partner formation and tertiary education. To date, the developmental marker of intimate relationship formation has been largely ignored in the literature. Instead, the extant literature has focused more on the importance of family and individual pro-social relationships in healthy development [27,40]. The importance of family, community, kinship, social networks, and interdependent relationships have been noted as contributing factors to the survival of Indigenous Peoples, despite ongoing colonial policies and practices [2]. Our findings strengthen the case for the importance of close relationships in the positive development of both Indigenous and non-Indigenous youth. Encouraging the formation of healthy close relationships may be warranted as a strategy to reduce inequalities and structural disadvantages that Indigenous Peoples continue to experience. Further research is required to better understand the protectiveness of intimate relationships for Indigenous and non-Indigenous youth.

Previous literature has shown how school completion can enhance healthy adult development [41], and provide a valuable space for delivering intervention and prevention programs [40]. Further findings demonstrate that school dropout can predict delinquent behavior [42]. There is also literature that highlights the use of scholarships, funded placements, and pathway programs to reduce structural inequalities among Indigenous and non-Indigenous youth within higher education [43]. Therefore, while higher education may be a useful tool to enhance positive development for some, additional support and resources should be made available. Indeed, our findings that Indigenous youth had a higher involvement in tertiary education may help encourage further exploration into the role of tertiary education as a protective factor for Indigenous and non-Indigenous youth. Importantly, when discussing the benefits of tertiary education, we must also acknowledge the complexity of expecting Indigenous Peoples to engage in mainstream education, which is primarily dominated by Western worldviews that undermine Indigenous knowledges [44]. While not within the scope of the current study, a more extensive discussion around the need to promote Indigenous education sovereignty is required [45]. We must further examine how to diminish structural disadvantages and utilize tertiary education involvement to enhance positive development among Indigenous youth. Similar suggestions have been made by Azzopardi and colleagues [46] who argue that through enhancing health outcomes among Indigenous adolescents, health equity on a larger scale may begin to be achieved.

The literature suggests that positive community experiences such as engaging with community members can help promote healthy development among young people [47]. The protectiveness of community engagement for young Indigenous Peoples has been previously reported in the USA [48] and Australia [49]. This literature postulates that greater engagement with, and positive influence from, the community is significantly associated with positive psychosocial functioning, resiliency, and positive adult development among young Indigenous Peoples [49]. The current findings, which show that community engagement is not differentially related to positive adult development for Indigenous youth, are consistent with these prior studies and extend them by adding a comparative analysis. Our findings suggest that enhancing community involvement is an important component of PYD programs for Indigenous and non-Indigenous youth. For Indigenous youth, such programs should be developed and implemented under the leadership of Indigenous communities, given their distinct cultural experiences and needs.

The findings of the present study demonstrate a positive association between volunteering and adult development. This is consistent with prior literature demonstrating that care-orientated behaviors and attitudes during adolescence significantly enhance positive development and adjustment in young adulthood [5,12,32]. Past studies, however, have not established whether this finding extends to youth from diverse backgrounds. Therefore, our study, which focuses on both Indigenous and non-Indigenous youth volunteer involvement, is particularly significant. Strachan and colleagues [50] provided some insight into the use of care to promote positive development among Indigenous youth in Canada. Participants highlighted the importance of care, including showing concern for others and caring for themselves (self-improvement) in their experience of positive development. Although there is a distinction between volunteering (spending time or money helping people or organizations) and caring behavior, both fit within the broader concept of care-orientated activities. Therefore, our findings add to the conclusions of Strachan and colleagues [50] and Hutchinson and colleagues [12] by demonstrating how care-orientated behavior can enhance PYD among both Indigenous and non-Indigenous youth. Of course, the ability to volunteer may be impacted by personal resources, socioeconomic status, race, and class [33]. Therefore, although our findings suggest that volunteer engagement may be a valuable tool to promote healthy development, we also acknowledge that some young people may have limited capacity or opportunity to volunteer. To overcome this to some extent, more opportunities to engage in volunteering and care-orientated activities at schools, in community groups, and within sports teams need to be created for all groups. Importantly, these initiatives should be Indigenous-led.

### Strengths and Limitations

The current study has several strengths, including a longitudinal design and large population samples of students surveyed using common measures. The IYDS used matched recruitment and survey methods cross-nationally, and the survey has demonstrated longitudinal and cross-national validity [36,51,52]. The use of pre-existing data also minimized the research burden on Indigenous communities.

There are some limitations of the current study which merit consideration when interpreting the findings. First and most importantly, both the IYDS and YPOF were designed by non-Indigenous researchers to assess health and social behavior among youth broadly, based on theories founded in Western, post-colonial approaches to science and knowledge. For example, we chose the PYD theory because of the lack of existing research on this framework with Indigenous youth, however other theories, including self-determination, may be preferred by some Indigenous communities [53]. We acknowledge that the PYD theory is unlikely to fully represent healthy development as experienced or understood by many Indigenous Peoples and communities. For example, although educational engagement and completion predict PYD and resilience for both Indigenous and non-Indigenous youth [18], as mentioned earlier, we must acknowledge the complexity of current education systems. Potential differences in the meaning of survey questions and other factors that may be relevant to Indigenous youth (opportunity to engage with Indigenous culture, discrimination, the ongoing impact of colonization) [7,18] were not measured. It is to be noted that, in the analysis of the data for this study, Indigenous researchers were consulted and have contributed to and co-authored this manuscript. Overall, positive development studies must consider measuring a range of alternative factors, including cultural engagement and discrimination.

Some other limitations should be noted. The PYD indicators were constructed from different measures in the IYDS and YPOF studies, reducing the sensitivity to examine important issues such as employment quality and satisfaction. Other aspects of individual context and personal characteristics (e.g., self-worth and positive identity) linked to the transition to adulthood were omitted here [54]. Gender was measured on a binary scale and did not capture transgender or non-gender-conforming individuals. Our study consisted of a sample of Australian youth drawn from Victoria, Western Australia, and Queensland, and a sample of youth drawn from Washington State, USA; the results may, therefore, not apply to youth more broadly in Australia and the USA. 

To assess the effect of missing data, chained regression equations generated 20 datasets using multiple imputation in STATA. The results suggested that findings were robust to missing data. The 95% confidence interval (CI) for the imputed model estimates (*N* = 14,496) encompassed the non-imputed estimates reported in Table 3, Model 3 (Age × Indigenous: Coefficient = −0.006, CI −0.011 to −0.002, *p* = 0.003. Community at age 15: Coefficient = 0.012, CI 0.006 to 0.018, *p* < 0.001. Indigenous x community at age 15: Coefficient = −0.003, CI −0.035 to 0.030, *p* = 0.877. Volunteering at age 15: Coefficient = 0.005, CI 0.002 to 0.009, *p* = 0.002. Indigenous x volunteering at age 15: Coefficient = −0.002, CI −0.018 to 0.015, *p* = 0.846).

## 5. Conclusions

The theory of PYD is often referred to when explaining or interpreting the healthy transition from adolescence to adulthood. While this theory has been unpacked and explored cross-nationally, the extant literature explores the theory among Indigenous youth to a limited extent. Indigenous Peoples globally continue to experience inequalities and disadvantages as promoted through colonization. While reducing these inequalities and disadvantages is an extensive process, we hope our study can contribute positively to this process by providing insight into predictors of positive development among Indigenous youth. However, while we have shown that volunteer and community engagement work similarly to promote healthy development for both Indigenous and non-Indigenous youth, we have not explored unique markers or predictors of development as understood by Indigenous communities. Therefore, we suggest that future research be conducted closely with Indigenous communities to reach an enhanced understanding of these unique predictors. We also recommend that schools, community organizations, and policymakers in the USA and Australia consider the possible usefulness of volunteer and community engagement in promoting healthy development.

We found that Indigenous youth showed slower increases in positive development over time compared to non-Indigenous youth. However, when controlling for socio-economic disadvantage, the difference in the growth of young adult development between Indigenous and non-Indigenous youth was reduced. Therefore, removing socioeconomic inequalities is crucial to support Indigenous youth. Further, the strength of association between volunteering and community engagement and positive adult development were not significantly different in the two groups. Through our findings, we support the arguments made by several researchers. These include Sanders and colleagues [18] who highlighted the importance of education engagement for Indigenous youth; Dudgeon and colleagues [51] who acknowledge the importance of pro-social relationships for Indigenous youth; Hawkins and colleagues [5] who revealed the protectiveness of volunteer involvement; and Kenyon and Carter [48], who reported on the protectiveness of community engagement.

There is an immediate need to work collaboratively with Indigenous Peoples and other marginalized communities to accurately recognize their needs and opinions regarding what is considered healthy and positive development. These conversations with community should guide future research and policy development. Such research will aid our understanding of how to enhance Indigenous youth development in line with a comprehensive set of factors relevant to Indigenous communities.

## Data Availability

We are unable to share our data publicly due to ethical reasons and confidentiality of the data.

## Appendix A

**Table A1:** Indigenous Status.

Variable	Study	Question	Response Options	Coding
Indigenous Status	YPOF	‘Are you or your family Aboriginal or Torres Strait Islander?’	Response options: Yes, No	Indigenous = yes (1) or non-Indigenous (0)
	IYDS (AUS)	‘What do you consider yourself to be?’	Response options: African, Aboriginal or Torres Strait Islander, Spanish, Asian, Pacific Islander, Australian.	Indigenous = Aboriginal or Torres Strait Islander (1) or non-Indigenous (0)
	IYDS (USA)	‘What best describes your racial background?’	Response options: white, black or African American, American Indian/Native American or Alaskan Native, Asian, Native Hawaiian or other Pacific Islander, Other	Indigenous = American Indian/Native American or Alaskan Native, Native Hawaiian or other Pacific Islander (1) or non-Indigenous (0)

**Table A2:** Dependent variables.

Variable	Study	Year	Wave	Average Age (Years)	Question	Response Options	Coding
Intimate relationship	YPOF	2019	Wave 2	17	Have you ever had sex?	0, No 1, Yes	Intimate relationship = 1
	IYDS (AUS)	2003	Wave 2	16			
	IYDS (USA)						
	IYDS (AUS)	2010	Wave 7	21	Are you currently or have your ever been in a romantic or intimate relationship?	1, Never 2, Yes, but not in the past 12 months 3, Yes in the past 12 months but not currently, 4. Yes, currently	Intimate relationship = 4
	IYDS (USA)						
	IYDS (AUS)	2012	Wave 8	23	Are you currently or have your ever been in a romantic or intimate relationship?	1, Never 2, Yes, but not in the past 12 months 3, Yes in the past 12 months but not currently, 4. Yes, currently	Intimate relationship = 4
	IYDS (USA)						
	IYDS (AUS)	2014	Wave 9	25	During the past 12 months, have you been in a romantic or intimate relationship?	−8, Not applicable −6, Refused −5, Logical skip 1, No 2, Yes, but not currently, 3, Yes, currently	Intimate relationship = 3
	IYDS(USA)						
	IYDS (AUS)	2018	Wave 10	29	What is your current marital status?	1, Single, never married 2, Engaged 3, Married 4, Living with partner, not married 5, Divorced 6, Widowed	Intimate relationship = 2, 3, or 4
	IYDS(USA)						
Year 12 completion	YPOF	2019	Wave 2	17	What is your schooling status?	1, In school 3, Home-school 5, Vocation school 6, University/college 7, TAFE 8, Working 9, Other	Year 12 completion = 6
	IYDS (AUS)	2003	Wave 2	16			
	IYDS (USA)						
	IYDS (AUS)	2010 2012 2014 2018	Wave 7 Wave 8 Wave 9 Wave 10	21 23 25 29	What is the highest level of secondary school you have completed?	1, Year 12 or equivalent 2, Year 11 or equivalent 3, Year 10 or equivalent 4, Year 9 or equivalent 5, Year 8 or below 6, Other	Year 12 completion = 1
	IYDS (USA)	2010 2012 2014 2018	Wave 7 Wave 8 Wave 9 Wave 10	21 23 25 29	What is the highest level of education you have completed?	1, Not applicable 2, Grade 9 3, Grade 10 4, Grade 11 5, Grade 12 6, GED certificate 7, Some technical or vocational school 8, Some college 9, Two-year college graduate 10, Four-year college graduate 11, Some postgraduate 12, Post college or professional degree 13, Other	Year 12 completion = 5, 6, 8 to 12
Tertiary (current or completed)	YPOF	2019	Wave 2	17	What year level are you in or what other kinds of studying are you doing?	1, Year 9 2, Year 10 3, Year 11 4, Year 12 5, Course at TAFE college, 6, Apprenticeship/Traineeship 7, Other	Tertiary education = 5, 6, or 7
	IYDS (AUS)	2003	Wave 2	16	What is your schooling status?	1, In school 3, Home-school 5, Vocation school 6, University/college 7, TAFE 8, Working 9, Other	Tertiary education = 6 or 7
	IYDS (USA)						
	IYDS (AUS) IYDS (USA)	2010	Wave 7	21	What courses or degrees have you studied since secondary school? What is the current status of this course/degree?	Apprenticeship/Traineeship Graduate diploma/Certificate Bachelor Degree Post Graduate Degree Still doing it, Completed, Deferred, Discontinued	Tertiary = Yes (1) to Still doing it or Completed any of the four course options.
	IYDS (AUS) IYDS (USA)	2012	Wave 8	23	What courses or degrees are you studying currently? What courses or degrees have you completed since secondary school?	Apprenticeship/Traineeship Graduate diploma/Certificate Bachelor Degree Post Graduate Degree Advanced Diploma/Diploma Certificate course Other Still doing it, Completed, Deferred, Discontinued	Tertiary = Yes (1) to Still doing it or Completed any of the four course options.
	IYDS (AUS)	2014 2019	Wave 9 Wave 10	25 29	What best describes your current educational status?	0, Not currently studying 1, Part-time student 2, Full-time student 3, Other	Tertiary = 1 or 2
	IYDS (USA)				What is the highest level of education you have completed, since secondary school?	0, Not applicable 1, Refused 2, Apprenticeship/Traineeship 3, Graduate diploma/certificate 4, Bachelor degree 5, Post graduate degree 6, Advanced Diploma/Diploma 7, Certificate course 8, Other 9, I have not completed study since secondary school	Tertiary = 2, 3, 4, 5, 6, or 7
Paid work	YPOF	2019	Wave 2	17	Are you working (in a paid job)?	1, No and I am not looking for work 2, No, but I am looking for work 3, Yes, I am working part-time/casually 4, Yes, I am working full-time	Paid work = 3 or 4
	IYDS (AUS)	2003	Wave 2	16	On average, over the school year, how many hours per week do you work in a paid job?	1, None 2, 5 or less 3, 6–10 4, 11–15 5, 16–20 6, 21–25 7, 26–30 8, 31 or more	Paid work = 2 or higher
	IYDS (USA)						
	IYDS (AUS)	2010 2012	Wave 7 Wave 8	21 23	What is your current source of income (paid employment)?	0, No 1, Yes	Paid work = 1
	IYDS (USA)						
	IYDS (AUS)	2014	Wave 9	25	What is your current source of income (paid employment)?	−8, Not applicable −7, Don’t know −6, Refused 0, No 1, Yes	Paid work = 1
	IYDS (USA)						
	IYDS (AUS)	2019	Wave 10	29	What is your current source of income (paid employment)?	0, No 1, Yes	Paid work = 1
	IYDS (USA)						
Independent income	YPOF	2019	Wave 2	17	During an average week, how much money do you get from a job or other work?	1, None 2, $1–$40 3, $41–$100 4, $101–$250 5, $251 or more	Independent income = 4 or 5
	IYDS (AUS)	2003	Wave 2	16	During an average week, how much money do you get from a job or other work?	1, None 2, 1$1–10 3, $11–20 4, $21–$35 5, $35–50 6, $51–75 7, $76–125 8, $126 or more	Independent income = 7 or 8
	IYDS (USA)						
	IYDS (AUS)	2010	Wave 7	21	What is your usual take home, weekly income from all sources of support?	1, $0 2, $1–$50 3, $51–$100 4, $101–$150 5, $151–$200 6, $201–$250 7, $251–$300 8, $301–$400 9, $401–$500 10, $501 or more	Independent income = 4, 5, 6, 7, 8, 9, or 10
	IYDS (USA)						
	IYDS (AUS)	2012	Wave 8	23	What is your usual take home, weekly income from all sources of support?	1, $0 2, $1–$50 3, $51–$100 4, $101–$150 5, $151–$200 6, $201–$250 7, $251–$300 8, $301–$400 9, $401–$500 10, $501-$600 11, $601–$800 12, $801–$1000 13, $1001 or more	Independent income = 4, 5, 6, 7, 8, 9, 10, 11, 12, or 13
	IYDS (USA)						
	IYDS (AUS)	2014 2019	Wave 9 Wave 10	25 29	What is your usual take home, weekly income from all sources of support?	1, $0 2, $1–$50 3, $51–$100 4, $101–$150 5, $151–$200 6, $201–$250 7, $251–$300 8, $301–$400 9, $401–$500 10, $501–$600 11, $601–$800 12, $801–$1000 13, $1001–$1200 14, $1201–$1400 15, $1401–$1600 16, $1601–$1800 17, $1801–$2000 18, $2001 or more	Independent income = 4 or more
	IYDS (USA)						

**Table A3:** Independent variables.

Variable	Study	Year	Wave	Average Age	Question	Response Options	Coding
Community involvement	YPOF	2017 2002 2004 2006	Wave 1 Wave 1 (oldest cohort) Wave 3 (middle cohort) Wave 4 (oldest cohort)	15 13 15 17	“There are people in my neighbourhood who are proud of me when I do something well” “There are people in my neighbourhood who encourage me to do my best” “My neighbours notice when I am doing a good job and let me know about it” “Which of the following activities for people your age are available in your community?’ Sports teams, scouting, youth groups, community service There are lots of adults in my neighbourhood I could talk to about something important	1, NO! 2, no 3, yes 4, YES!	Responses from the 4 items were averaged to form an overall community involvement scale (range 1–4)
	IYDS (AUS) IYDS (USA)						
Volunteering	YPOF	2017	Wave 1	15	“How often have you done volunteer work to help other people, such as helping out as hospital or raising money for charity?”	1, Never 2, Once 3, Twice 4, 3–4 times 5, More than 5 times	Overall volunteering was recoded on a scale from 1–4
	IYDS (AUS)	2002 2004 2006	Wave 1 (oldest cohort) Wave 3 (middle cohort) Wave 4 (oldest cohort)	13 15 17	“In the past 12 months have you spent any time doing voluntary work through an organisation or group?” “How often in the past 12 months did you volunteer?” “How often have you done volunteer work to help other people, such as helping out as hospital or raising money for charity?”.	1, Never 2, Once 3, Twice 4, 3–4 times 5, More than 5 times	The items were combined, and overall volunteering was recoded on a scale from 1–4
	IYDS (USA)						

**Table A4:** Control variables.

Variable	Study	Year	Wave	Average Age	Question	Response Option	Coding
Parent education	YPOF	2017	Wave 1	15	What is your mother’s/female guardian’s highest level of education? What is your father’s/male guardian’s highest level of education?	0, I don’t know 1, Didn’t complete high school (year 12) 2, Completed high school (year 12) 3, Has a degree from a university	Education level averaged across both parents and coded on 3-point scale. Completed (1) less than secondary school, (2) secondary school, (3) post-secondary school
	IYDS (AUS)	2002 2004 2006	Wave 1 (oldest cohort) Wave 3 (middle cohort) Wave 4 (oldest cohort)	13 15 17	Parent report of highest education completed for both parents	Grade 7 or less; Grade 8; Grade 9; Grade 10; Grade 11; GED; High School Graduate/Year 12; Some Trade or Business School; AA Degree; Some College; College Graduate; Postgraduate Degree (MA/PHD); Other (specify)	Education level averaged across both parents Completed (1) less than secondary school, (2) secondary school, (3) post-secondary
	IYDS (USA)						
Government School	YPOF	2017	Wave 1	15	School ID number	Not applicable	Participants were categorised into a school classification based on school ID. We recoded this to be (1) Public or Government school or (0) Independent private, Religious private, Independent, or Catholic.
	IYDS (AUS)	2002 2004 2006	Wave 1 (oldest cohort) Wave 3 (middle cohort) Wave 4 (oldest cohort)	13 15 17	School ID number	Not applicable	Participants were categorised into a school classification based on school ID. We recoded this to be (1) Public or Government school or (0) Independent private, Religious private, Independent, or Catholic.
	IYDS (USA)						
Urbanicity	YPOF	2017	Wave 1	15	School location (local government area)	Not applicable	LGA was coded as either urban (1) or regional (2) based on the Australian Bureau of Statistics
	IYDS (AUS)	2002 2004 2006	Wave 1 (oldest cohort) Wave 3 (middle cohort) Wave 4 (oldest cohort)	13 15 17	School location (local government area)	Not applicable	LGA was coded as either urban (1) or regional (2) based on the Australian Bureau of Statistics
	IYDS (USA)	2002 2004 2006	Wave 1 (oldest cohort) Wave 3 (middle cohort) Wave 4 (oldest cohort)	13 15 17	School location (sector)	Not applicable	Categories used were urban and urban fringe, large and small towns, and rural areas. These categories were based upon the US National Center for Education Statistics school-level definitions of urban city, towns, and rural areas. We recoded this as (1) urban or (2) regional.

## References

[1] Aguiar W., Halseth R. (2015). Aboriginal Peoples and Historic Trauma: The Processes of Intergenerational Transmission.

[2] Paradies Y. (2020). Unsettling truths: Modernity, (de-)coloniality and Indigenous futures. Postcolonial Stud..

[3] Priest N., Perry R., Ferdinand A., Kelaher M., Paradies Y. (2017). Effects over time of self-reported direct and vicarious racial discrimination on depressive symptoms and loneliness among Australian school students. BMC Psychiatry.

[4] Walker R., Sonn C., Dudgeon P., Milroy H., Walker R. (2020). Mental Health Practice. Working Together: Aboriginal and Torres Strait Islander Mental Health and Wellbeing Principles and Practice.

[5] Hawkins M.T., Letcher P., Sanson A., O’Connor M., Toumbourou J., Olsson C. (2011). Stability and change in positive development during young adulthood. J. Youth Adolesc..

[6] Bruner M.W., Hillier S., Baillie C.P.T., Lavallee L.F., Bruner B.G., Hare K., Lovelace R., Lévesque L. (2016). Positive Youth Development in Aboriginal Physical Activity and Sport: A Systematic Review. Adolesc. Res. Rev..

[7] Deane K., Dutton H., Kerekere E. (2019). Ngā Tikanga Whānaketanga He Arotake Tuhinga: A Review of Aotearoa New Zealand Youth Development Research.

[8] Cuervo H., Barakat N., Turnbull M. (2015). Youth, Belonging and Transitions: Identifying Opportunities and Barriers for Indigenous Young People in Remote Communities.

[9] Sittner K.J., Estes M.L. (2020). Adult outcomes of justice involved Indigenous youth. Race Justice.

[10] Lerner R.M., Agans J., Arbeit M.R., Chase P.A., Weiner M.B., Schmid K.L., Warren A.E.A. (2013). Resilience and positive youth development: A relational developmental systems model. Handbook of Resilience in Children: Second Edition.

[11] Catalano R.F., Berglund L.M., Ryan J.A.M., Lonczak H.S., Hawkins D.J. (2004). Positive youth development in the United States: Research findings on evaluations of positive youth development programs. Annals Am. Acad. Political Soc. Sci..

[12] Hutchinson D., Youssef G.J., Buhagiar A., Teague S., Maconald J.A., Letcher P., Greenwood C., McIntosh J., Toumbourou J.W., Hallam B.W.T. (2019). Adolescent care-orientation and positive development in young adulthood. J. Adolesc. Health.

[13] Wiium N. (2019). Dimitrova Positive youth development across cultures: Introduction to the special issue. Child Youth Care Forum.

[14] Langham E., McCalman J., Matthews V., Bainbridge R.G., Nattabi B., Kinchin I., Bailie R. (2017). Social and Emotional Wellbeing Screening for Aboriginal and Torres Strait Islanders within Primary Health Care: A Series of Missed Opportunities?. Front. Public Health.

[15] Wexler L. (2009). The Importance of Identity, History, and Culture in the Wellbeing of Indigenous Youth. J. Hist. Child. Youth.

[16] Galliher R.V., Jones M.D., Dahl A. (2011). Concurrent and longitudinal effects of ethnic identity and experiences of discrimination on psychosocial adjustment of Navajo adolescents. Dev. Psychol..

[17] Snowshoe A., Crooks C.V., Tremblay P.F., Hinson R.E. (2017). Cultural connectedness and its relation to mental wellness for First Nations youth. J. Prim. Prev..

[18] Sanders J., Munford R., Liebenberg L. (2017). Positive youth development practices and better outcomes for high risk youth. Child Abus. Negl..

[19] Hishinuma E., Chang J.Y., Sy A., Greaney M.F., Morris K.A., Scronce A.C., Rehuher D., Nishimura S.T. (2009). HUI Malama O Ke Kai: A positive prevention-based youth development program based on native Hawaiian values and activities. J. Community Psychol..

[20] Dockery A.M. (2020). Inter-generational transmission of Indigenous culture and children’s wellbeing: Evidence from Australia. Int. J. Intercult. Relat..

[21] McDonough M.H., Ullrich-French S., McDavid M.L. (2017). Helping kids connect: Participant and staff perspectives on facilitating social relationships in physical activity-based positive youth development program for youth from low-income families. Sport Exerc. Perform. Psychol..

[22] Orejudo S., Balaguer A., Osorio A., de la Rosa P.A., Burgo C.L. (2021). Activities and relationships with parents as key ecological assets that encourage personal positive youth development. J. Community Psychol..

[23] Arnett J.J. (2007). Emerging adulthood: What is it, what is it good for?. Child Dev. Perspect..

[24] Oesterle S., Hill K.G., Hawkins J.D., Abbott R.D. (2008). Positive functioning and alcohol-use disorders from adolescence to young adulthood. J. Stud. Alcohol Drugs.

[25] Crooks C.V., Exner-Corten D., Burm S., Lapointe A., Chiodo D. (2016). Two years of relationship-focused mentoring for First Nations, Metis, and Inuit adolescents: Promoting positive mental health. J. Prim. Prev..

[26] Brave Heart M.Y., Chase J., Elkins J., Altschul D.B. (2011). Historical trauma among Indigenous Peoples of the Americas: Concepts, research, and clinical considerations. J. Psychoact. Drugs.

[27] Allen J., Hopper K., Wexler L., Kral M., Rasmus S., Nystad K. (2014). Mapping resilience pathways of Indigenous youth in five circumpolar communities. Transcult. Psychiatry.

[28] Pu J., Chewing B., St Clair L.D., Kokotailo P.K., Lacourt J., Wilson D. (2013). Protective factors in American Indian Communities and adolescent violence. Matern. Child Health J..

[29] Lowe K., Harrison N., Tennent C., Guenther J., Vass G., Moodie N. (2019). Factors affecting the development of school and Indigenous community engagement: A systematic review. Aust. Educ. Res..

[30] Sayers J.M., Hunt G.E., Cleary M., Burmeister O.K. (2016). Brokering Community Engagement: Proactive Strategies for Supporting Indigenous Australians with Mental Health Problems. Issues Ment. Health Nurs..

[31] LaFromboise T.D., Hoyt D.R., Oliver L., Whitbeck L.B. (2006). Family, community, and school influences on resilience among American Indian adolescents in the upper midwest. J. Community Psychol..

[32] Hutchinson D., Macdonald J.A., Hallam W.T., Leung R.K., Toumbourou J.W., McGee R., Tooley G., Hemphill S.A., Skouteris H., Olsson C.A. (2016). Care orientation in the teens as a predictor of young adult psychosocial adjustment. J. Happiness Stud..

[33] Musick M.A., Wilson J., Bynum W.B. (2000). Race and Formal Volunteering: The Differential Effects of Class and Religion. Soc. Forces.

[34] Heerde J.A., Bailey J.A., Toumbourou J.W., Rowland B., Catalano R.F. (2022). Adolescent antecedents of young adult homelessness: A cross-national pathway analysis. Prev. Sci..

[35] Kish L. (1965). Survey Sampling.

[36] McMorris B.J., Hemphill S.A., Toumbourou J.W., Catalano R.F., Patton G.C. (2006). Prevalence of substance use and delinguent behaviour in adolescents from Victoria, Australia and Washington State, United States. Health Educ. Behav..

[37] Rowland B., Jonkman H., Steketee M., Solomon R.J., Solomon S., Toumbourou J.W. (2021). A Cross-National Comparison of the Development of Adolescent Problem Behavior: A 1-Year Longitudinal Study in India, the Netherlands, the USA, and Australia. Prev. Sci..

[38] Bodkin-Andrews G., Carlson B. (2014). The legacy of racism and Indigenous Australian identity within education. Race Ethn. Educ..

[39] Solomon T.G.A., Starks R.R.B., Attakai A., Molina F., Cordova-Marks F., Kahn-John M., Antone C.L., Flores M., Garcia F. (2022). The generational impact of racism on health: Voices from American Indian communities. Health Equity.

[40] Young C., Tong A., Nixon J., Fernando P., Kalucy D., Sherriff S., Clapham K., Craig J.C., Williamson A. (2017). Perspectives on childhood resilience among the Aboriginal community: An interview study. Aust. N. Z. J. Public Health.

[41] Freudenberg N., Ruglis J. (2007). Reframing school dropout as a public health issue. Prev. Chronic Dis..

[42] Henry K.L., Knight K.E., Thornberry T.P. (2012). School disengagement as a predictor of dropout, delinquency, and problem substance use during adolescence and early adulthood. J. Youth Adolesc..

[43] Rendon A.L., Al-Asfour A. (2019). Lakota female scholarship: A collective case study to transcending Indigenous educational pathways and persistence at the graduate level. J. Educ. Issues.

[44] Yunkaporta T. (2019). Sand Talk: How Indigenous Thinking Can Save the World.

[45] Bishop M. (2021). A rationale for the urgency of Indigenous education sovereignty: Enough’s enough. Aust. Educ. Res..

[46] Azzopardi P., Blow N., Purcell T., Brown N., Ritchie T., Brown A. (2020). Inveting in the health of Aboriginal and Torres Strait Islander adolescents: A foundation for achieving health equity. Med. J. Aust..

[47] Toumbourou J.W. (2016). Beneficial action within altruistic and pro-social behaviour. Rev. Gen. Psychol..

[48] Kenyon D.B., Carter J.S. (2011). Ethnic identity, sense of community, and psychological well-being among northern plains American Indian youth. J. Community Psychol..

[49] Hopkins K.D., Shepherd C.C., Taylor C.L., Zubrick S.R. (2015). Relationships between psychosocial resilience and physical health status of Western Australian urban Aboriginal youth. PLoS ONE.

[50] Strachan L., McHugh T., Mason C. (2018). Understanding positive youth development in sport through the voices of Indigenous youth. J. Sport Exerc. Psychol..

[51] Heerde J.A., Bailey J.A., Toumbourou J.W., Rowland B., Catalano R.F. (2020). Longitudinal associations between early-mid adolescent risk and protective factors and young adult homelessness in Australia and the United States. Prev. Sci..

[52] Toumbourou J.W., Evans-Whipp T.J., Smith R., Hemphill S.A., Herrenkohl T.I., Catalano R.F. (2014). Adolescent predictors and environmental correlates of young adult alcohol use problems. Addiction.

[53] Dudgeon P., Blustein S., Bray A., Calma T., McPhee R., Ring I. (2021). Connection between family, kinship and social and emotional wellbeing. Produced for the Indigenous Mental and Suicide Prevention Clearninghouse.

[54] Geldhof G.J., Larsen T., Urke H., Holsen I., Lewis H., Tyler C.P. (2019). Indicators of positive youth development can be maladaptive: The examples of caring. J. Adolesc..

